# Glances at the Insane in Other Lands

**Published:** 1889-03-09

**Authors:** 


### GLANCES AT THE INSANE IN OTHER LANDS.

BY A MEDICAL SUPERINTENDENT.

(Continued from p. 353.)

### VI.—VICTORIA.

EVEN at the antipodes the babel of voices rises over the ever ending asylum question, and the *Melbourne Argus* has had its columns filled with reports of papers read at medical societies; with leading articles, with debates, and with long letters pseudonymous and otherwise. Here is the charge made in the *Argus* of November 28, 1888, over the high-sounding name of Telemachus—"You have in the asylums of Victoria upwards of 500 more patients, men and women, than you have adequate and healthful provision for. You have taken the whole business of providing for lunatics into your own hands. You have forbidden private enterprise to meddle with it. You are responsible, therefore, for the safe keeping and proper treatment of every human being declared insane in the colony. Now, what have you done? We declare that you have overcrowded all your asylums; that you have not carried out any proper system of classification; that you have been guilty of grievous wrong against patients in the first stages of insanity by confining them with confirmed and hopeless lunatics. Further, you have not studied sanitary laws in the building, or equipping of your asylums. You are exercising, indeed, a parsimonious thrift, and committing a grievous wrong, and imposing a debt on the country that will not be paid through a century's laborious performance of duty. Your Kew is a mistake; your Yarra Band a disgrace; and instant and vigorous and enthusiastic effort is necessary to place the whole matter on a satisfactory basis, and purge us of our self-shame and external contempt." This is a terrible indictment, and it is given as the substance of a speech delivered in the Victorian Parliament. While something must be allowed for statements made in a speech, there would seem to be no doubt that the insane of the colony are insufficiently provided for, and that many reforms are imperatively necessary before the Victorians can claim that their lunatics are properly cared for. There is apparently a consensus of opinion among all parties concerned, that it is time something was done; but, unfortunately, there is not perfect accord as to what that something should be—one section being convinced that the cottage system of asylum construction is the best, and the other adhering to some modification of the so-called barrack system. In whichever way this point may ultimately be settled, there is one thing certain—that is, where the Board of Management of an asylum does not anticipate the wants of the district, all experience shows that the accommodation will lay behind it. Now, there is no excuse for this, because the increase in the numbers of the insane for any district is a fairly well-known quantity, and it is surely more business-like, and generally more economical also, to build additions or new asylums in time, rather than try and postpone for a year or two what must be done in any case. Telemachus truly says, that reform in asylums should come before exhibitions and picture galleries, and Parliament houses.

The *Argus* thinks that one thing wanted is a better classification of cases suitable for asylums; and that another is a large increase in the accommodation for lunatics. It is also suggested that the physicians certifying to the insanity of a patient should have special knowledge of the subject. This would be a very excellent thing, but as we have not seen our way to effect it on this side of the world, we doubt whether it will be easily done in Australia, but we should be glad to see it tried.

It has been suggested to sell the Yarra Band site and build new asylums in other and more distant parts of the colony. While a colony of eighty-eight thousand square miles, and a million of inhabitants, ought to have its institutions for the insane in convenient places, it is clear that its chief one ought to be close to Melbourne, and should be affiliated with the Melbourne University for teaching purposes.

The authority who inspires the *Argus* in asylum matters prefers the cottage system to the barrack; but we are inclined to agree with Dr. Barker, who, in his address to the Medical Society of Melbourne, showed that the expense of construction of the cottage system would be greater; that the difficulty of supervision of patients would be greater, and also the difficulty of looking after the staff. In fact, the whole system is surrounded with difficulties; but only one need be insisted on, as it constitutes the barrier to the whole position, and that is the impossibility of finding cases suitable for treatment in cottages. Dr. Manning, the greatest authority in Australia on asylum matters, gives it as his opinion that not more than ten per cent. of the inmates of asylums can be safely treated in cottages; and this calculation, modest as it is, is probably above the mark. In one asylum with which we are acquainted there is a detached building for twenty-four females. With a total number of nearly four hundred women in the asylum, it is almost impossible to keep the detached building full of suitable cases—that is, cases who would do better there than in the main building. At first sight there is something captivating about the cottage system; but when weighed in the balance it is found wanting in the requirements of a system, and takes a very subordinate position as a rather pleasant, but not very important, adjunct to the "barracks." If those who think otherwise will reckon up the epileptics, the suicidal cases, the homicidal, the acute and subacute maniacs, the sick, the general paralytics, and some other classes which need not be specially referred to here, it would be found that the number remaining is too small to necessitate any departure from the old plan. Nevertheless, we are of opinion that two detached buildings, one for men and one for women, would be desirable, such buildings being able to accommodate about five per cent. of the respective sexes.

In one of the papers it is stated that the power given to the medical superintendents of Victorian asylums is too limited. This is a fatal mistake. Let the medical superintendent have both power and responsibility. Where there is insufficient power given it must mean that the Board undertakes more or less of the management. In other words, the board, which ought to be legislative only, becomes the executive — a system which is alike fatal to institutions and to nations, and peremptorily calls for reform. It would seem that the head attendants and head nurses are, in some of the asylums, appointed to these extremely responsible posts merely on grounds of seniority. Now, a man may be a very good charge attendant, and not a very good head attendant, and to discharge the duties of the latter well much energy and activity are imperative, and also an intelligence and education superior to the average run of attendants. A liberal pension scheme would do away with any excuse for the promotion of unsuitable people; and it would bring and retain good officials to all departments.

The Victorians are now in a position to build an asylum which might form a model for the whole of Australia, perhaps for England too. It is earnestly to be wished that they will limit the number of beds to eight hundred, or a thousand at the very most, and that detached buildings will be few and small.

